# Reduced RBM3 expression is associated with aggressive tumor features in esophageal cancer but not significantly linked to patient outcome

**DOI:** 10.1186/s12885-018-5032-z

**Published:** 2018-11-12

**Authors:** Katharina Grupp, Bianca Hofmann, Asad Kutup, Kai Bachmann, Dean Bogoevski, Nathaniel Melling, Faik Guntac Uzunoglu, Alexander Tarek El Gammal, Christina Koop, Ronald Simon, Stefan Steurer, Till Krech, Susanne Burdak-Rothkamm, Frank Jacobsen, Guido Sauter, Jakob Izbicki, Waldemar Wilczak

**Affiliations:** 1 Institute of Pathology, University Medical Center Hamburg-Eppendorf, Hamburg, Germany; 2 General, Visceral and Thoracic Surgery Department and Clinic, University Medical Center Hamburg-Eppendorf, Hamburg, Germany

**Keywords:** RBM3, Tissue microarray, Prognosis, Esophageal cancer

## Abstract

**Background:**

RBM3 expression has been suggested as prognostic marker in several cancer types. The purpose of this study was to assess the prevalence and clinical significance of altered RBM3 expression in esophageal cancer.

**Methods:**

RBM3 protein expression was measured by immunohistochemistry using tissue microarrays containing samples from 359 esophageal adenocarcinoma (EAC) and 254 esophageal squamous cell cancer (ESCC) patients with oncological follow-up data.

**Results:**

While nuclear RBM3 expression was always high in benign esophageal epithelium, high RBM3 expression was only detectable in 66.4% of interpretable EACs and 59.3% of ESCCs. Decreased RBM3 expression was linked to a subset of EACs with advanced UICC stage and presence of distant metastasis (*P* = 0.0031 and *P* = 0.0024). In ESCC, decreased RBM3 expression was associated with advanced UICC stage, high tumor stage, and positive lymph node status (*P* = 0.0213, *P* = 0.0061, and *P* = 0.0192). However, RBM3 expression was largely unrelated to survival of patients with esophageal cancer (EAC: *P* = 0.212 and ESCC: *P* = 0.5992).

**Conclusions:**

In summary, the present study shows that decreased RBM3 expression is associated with unfavourable esophageal cancer phenotype, but not significantly linked to patient prognosis.

## Background

Esophageal cancer is one of the most aggressive cancers and is the sixth leading cause of cancer death worldwide. Currently, there are limited clinical approaches for the early diagnosis and treatment of esophageal cancer, resulting in only a 10% five-year survival rate for patients. It can be hoped, that the identification of novel biological markers and tumorigenic pathways will improve therapeutic strategies for esophageal cancer patients.

RBM3 is a glycine-rich RNA-binding protein and part of the family of cold shock proteins [1]. Proteins from this family are induced by various environmental stresses, including hypoxia [2] and cold [2–4]. RBM3 bind to DNA and RNA [5] and is involved in maintenance of DNA integrity, including DNA-dependent replication, DNA replication, chromatin remodeling, DNA integrity checkpoints [6] and regulation of RNA metabolism, including splicing, stability and transport of mRNA [1].

In cancer there are multiple contradictory reports regarding the role and expression of RBM3. RBM3 has been suggested to have potential proto-oncogene and tumor suppressive roles in cancer. RBM3 expression leads to the synthesis of proteins associated with survival and proliferation [7–9] and to the synthesis of proteins associated with apoptosis [10]. Using immunohistochemistry, dysregulated RBM3 expression has been suggested as prognostic biomarker in several cancers [11–19].

Only one immunohistochemical study has so far analysed RBM3 expression in upper gastrointestinal adenocarcinomas using a tissue microarray containing of 175 tumor specimens [12]. The authors suggested a potential prognostic role of altered RBM3 expression in patients with upper gastrointestinal adenocarcinomas [12]. To better assess the role of RBM expression in esophageal cancers, immunohistochemistry was applied to tissue microarrays containing samples from more than 600 esophageal cancer patients with oncological follow-up data. The present study shows that decreased RBM3 expression is linked to a subset of esophageal cancers with unfavourable tumor phenotype.

## Methods

### Patients, follow-up, and TMA construction

Two TMA were constructed from cancer tissues from 359 EAC and 254 ESCC patients who underwent surgery at the University Medical Center Hamburg-Eppendorf. Follow-up data were available of 359 EAC and 254 ESCC patients with a median follow-up of 17.3 and 12.2 months (range: 0 to 208 and 0 to 191 months). All esophageal specimens were analyzed according to a standard procedure, including complete embedding of the entire esophagus for histological analysis. The TMA manufacturing process was described earlier in detail [20]. In short, one 0.6 mm core was taken from a representative tissue block from each patient. The tissues were distributed among 2 TMA blocks. For internal controls, each TMA block also contained various control tissues, including normal esophageal tissue.

### Immunohistochemistry

For Immunohistochemistry, slides were deparaffinized and exposed to heat-induced antigen retrieval for 5 min in an autoclave at 121 C in pH 7.8 Tris- EDTA- Citrate buffer. Primary antibody specific for RBM3 (polyclonal rabbit, HPA003624; Sigma–Aldrich; at 1/150 dilution) was applied at 37 °C for 60 min. Bound antibody was then visualized using the EnVision Kit (Dako, Glostrup, Denmark) according to the manufacturer instructions. Since nuclear staining was typically paralleled by similar or slightly lower cytoplasmic staining, only nuclear staining was considered. For statistical analyses, the staining results were categorized in two groups: low and high RBM3 immunostaining.

### Statistical analysis

Statistical calculations were done with JMP® software (SAS Institute Inc., NC, USA). Contingency tables were performed to search for associations between molecular parameters and tumor phenotype. Chi-square (Likelihood) test was employed to identify significant relationships between these parameters. Survival curves were calculated according to Kaplan–Meier. Log-Rank test was applied to test for significant differences between stratified survival curves. Cox proportional hazards regression analysis was calculated to test the statistical independence and significance between pathological, molecular and clinical variables.

## Results

### RBM3 immunostaining in esophageal cancers

**Fig. 1:**
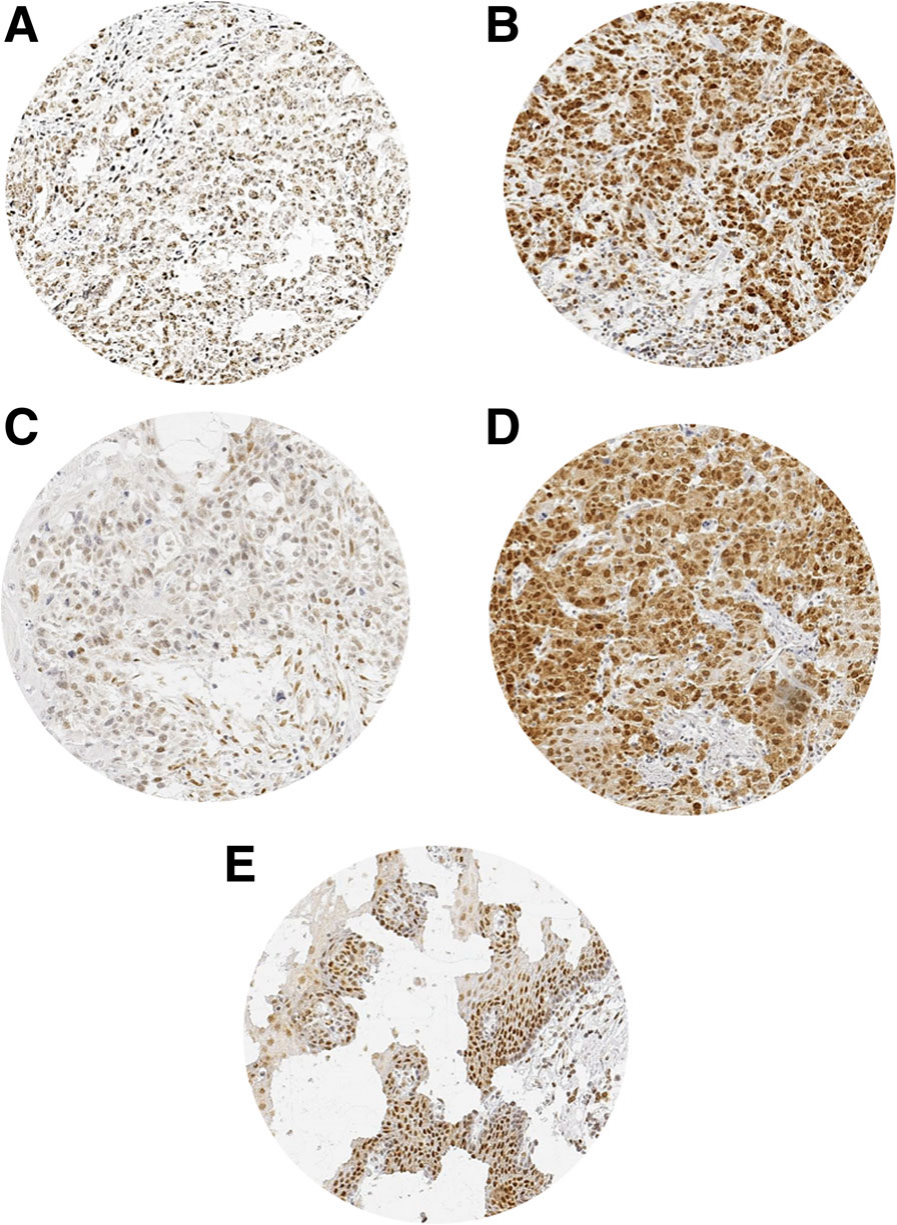
Representative images of RBM3 immunostaining in oesophageal cancer. The image shows low and high RBM3 immunostaining in EACs (**a** and **b**) and ESCCs (**c** and **d**). Image of RBM3 immunostaining in benign squamous esophageal tissue (**e**)

A total of 268 of 359 (74.7%) of EAC and 226 of 254 (89%) of ESCC samples were interpretable for immunohistochemistry in our TMA analysis. Reasons for non-informative cases included a complete lack of tissue samples or absence of unequivocal cancer tissue in the TMA section. RBM3 expression was high in benign esophageal epithelium predominantly localized in the nucleus of the cells. The expression levels of RBM3 were homogenous in the analyzed spots. RBM3 expression was found in decreased intensities in malignant as compared to benign esophageal epithelium. In malignant oesophagus, high RBM3 expression was only detectable in 66.4% of 268 interpretable EAC and in 59.3% of 226 ESCC specimens. Figure 1 shows representative pictures of RBM3 immunostaining in esophageal cancers and benign squamous esophageal tissue.

### Association with tumor phenotype and clinical outcome

The associations between RBM3 immunostaining and tumor phenotype are depicted in Tables 1 and 2. Decreased RBM3 expression was linked to a subset of EACs with advanced UICC stage and presence of distant metastasis (*P* = 0.0031 and *P* = 0.0024). In ESCC, decreased RBM3 expression was associated with advanced UICC stage, high tumor stage, and positive lymph node status (*P* = 0.0213, *P* = 0.0061, and *P* = 0.0192).

**Table 1:** Association of RBM3 IHC results and clinico-pathological features of esophageal adenocarcinoma samples

	RBM3 immunohistochemistry			
	Analyzable, n	Low, %	High, %	*p* value
All cancers	268	33.58	66.42	
Age group				
< 65 years	84	65.76	34.24	0.7355
> 65 years	184	67.86	32.14	
Sex				
male	226	33.63	66.37	0.9703
female	42	33.33	66.67	
Tumor stage				
pT1	55	23.64	76.36	0.333
pT2	28	35.71	64.29	
pT3	163	36.81	63.19	
pT4	20	35	65	
UICC stage				
I	52	19.23	80.77	0.0031
II	36	30.56	69.44	
III	155	34.84	65.16	
IV	24	62.5	37.5	
Tumor grading				
G1	14	21.43	78.57	0.6477
G2	98	35.71	64.29	
G3	150	33.33	66.67	
G4	4	50	50	
Resektion margin				
R0	193	34.72	65.28	0.4129
R1	69	30.43	69.57	
R2	3	66.67	33.33	
Lymph node metastasis				
N0	82	24.39	75.61	0.1623
N1	43	34.88	65.12	
N2	65	41.54	58.46	
N3	75	34.67	65.33	
Distant metastasis				
M0	244	30.74	69.26	0.0024
M1	24	62.5	37.5

**Table 2:** Association of RBM3 IHC results and clinico-pathological features of esophageal squamous cell carcinoma samples

	RBM3 immunohistochemistry			
	Analyzable, n	Low, %	High, %	*P* value
All cancers	226	40.71	59.29	
Age group				
< 65 years	86	46.51	53.49	0.178
> 65 years	139	37.41	62.59	
Sex				
male	160	41.88	58.13	0.6362
female	65	38.46	61.54	
Tumor stage				
pT1	40	17.5	82.5	0.0061
pT2	46	41.3	58.7	
pT3	126	46.83	53.17	
pT4	14	50	50	
UICC stage				
I	55	23.64	76.36	0.0213
II	57	45.61	54.39	
III	102	47.06	52.94	
IV	10	50	50	
Tumor grading				
G1	3	33.33	66.67	0.3216
G2	141	44.68	55.32	
G3	81	34.57	65.43	
G4	0	0	0	
Resektion margin				
R0	168	39.29	60.71	0.0717
R1	47	40.43	59.57	
R2	9	77.78	22.22	
Lymph node metastasis				
N0	100	35	65	0.0192
N1	55	32.73	67.27	
N2	40	55	45	
N3	29	58.62	41.38	
Distant metastasis				
M0	216	59.72	40.28	0.366
M1	9	44.44	55.56	

**Fig. 2:**
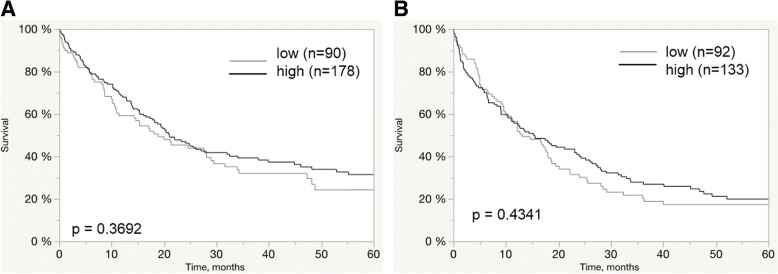
Clinical impact of RBM3 immunostaining. Relationship of RBM3 immunostaining with overall survival in EACs (*P* = 0.212; **a**) and ESCCs (*P* = 0.5992; **b**)

Follow-up data were available for 173 EAC and 166 ESCC patients with informative RBM3 data. RBM3 expression was not associated with overall survival of EAC and ESCC patients (*P* = 0.212 and *P* = 0.5992). The relationship between RBM3 immunostaining intensity and clinical outcome of the patients is shown in Fig. 2.

### Association between RBM3 expression and p53 expression in esophageal cancers

To evaluate whether RBM3 expression is linked to p53 expression in esophageal cancer, our pre-existing database including data on p53 immunostaining (unpublished data) was used. Our results demonstrate that RBM3 expression was unrelated to p53 expression in all esophageal cancer samples (*p* = 0.1098), as well as the subset of EACs (*p* = 0.8339) and ESCCs (*p* = 0.1466).

## Discussion

The data from this study show, that altered RBM3 expression – a strong prognostic feature in several cancer types – is statistically linked to adverse tumor features in oesophageal cancer but does not have an obvious impact on clinical outcome.

The comparative analysis of normal and neoplastic epithelium revealed that RBM3 expression was reduced in a fraction of cancers. These results are in line with data of one earlier study analyzing RBM3 expression in 60 EAC samples [12]. The authors also found a significantly higher RBM3 expression in normal squamous epithelium as compared to primary tumours [12].

Earlier studies on RBM3 expression in other malignancies have often indicated a biological and clinical significance of RBM3 expression, but results varied substantially between tumor entities. An increase of RBM3 in malignant relative to corresponding benign tissue has been suggested in gastric [12], prostate [11, 17] and breast [19] cancer. However, a decreased RBM3 expression in cancerous relative to non-cancerous tissue has been reported in colon cancer [21], malignant melanoma [16], and urothelial bladder cancer [14]. These discrepant observations may be due to different interactions between RBM3 and other gene products expressed and activated in individual tissue types.

In vitro studies on the role of RBM proteins in tumorigenesis have also yielded conflicting results and the exact function of RBM3 is still to be fully elucidated. So far, pro-oncogenic as well as tumor suppressive functions of RBM3 have been suggested [6, 10, 22, 23]. Some studies suggested RBM3 to induce cell proliferation and inhibit the DNA damage response machinery and cell death [6, 22, 23], while others suggested RBM3 to be positive associated with the pro-apoptotic BAX gene [10], to be downregulated in an in vitro model of melanoma progression [24], and to enhance platinum-sensitivity in vitro in ovarian cancer [18]. Our finding of reduced RBM3 expression being associated with advanced tumor stage in adenocarcinoma of the esophagus is supported by findings of Jonsson et al. [12] describing a significant association between reduced RBM3 expression and a more aggressive tumor phenotype. Our data further suggest, that RBM3 might play a comparable role in squamous cell carcinomas. In this subset of esophageal cancers, reduced RBM3 expression was statistically linked to adverse tumor features. The fact that reduced RBM3 expression was linked to development of cancer and was associated with adverse tumor features in our study suggests a tumor suppressive rather than an oncogenic role in esophageal cancers. Previously, studies have shown that BAX also plays a critical role in the determination of tumor response to radiation therapy in esophageal carcinoma cells [25]. Thus, it can be speculated that RBM3 might be also associated to the expression of pro-apoptotic BAX gene in our study. However, the exact mechanism how reduced RBM3 dives esophageal carcinogenesis remains elusive.

However, in our study, RBM3 expression loss has no clinically relevant impact on patient prognosis in both entities. These data disagree with the study of Jonsson et al. [12] suggesting RBM3 expression as a relevant prognostic marker in a mixed cohort of gastric and esophageal adenocarcinomas. These discrepant results may be due to differences in the patient cohorts. We only included esophageal cancer patients in our study. It can be speculated that the prognostic impact of RBM3 expression in the Jonsson’s mixed cohort of upper gastrointestinal adenocarcinomas, was largely driven by the subset of gastric cancers.

To evaluate whether RBM3 expression is linked to p53 expression in esophageal cancer, our pre-existing database on p53 immunostaining was used. In general, the pathogenesis of esophageal carcinoma is highly complex, involving an accumulation of genetic modifications resulting in invasive carcinoma [26, 27]. Genetic mutations within the tumour suppressor gene, *TP53,* which is involved in DNA repair and cell cycle arrest [26, 28, 29], is one of the most frequently genetic abnormalities in cancers [30]. Thus, we next correlated RBM3 expression with pre-existing data on p53 IHC in esophageal cancer. Our results demonstrate that RBM3 expression was unrelated to p53 expression in esophageal cancer samples.

Previously, authors speculated that the analysis of significances between biomarkers and clinical outcome is limited due to tumour heterogeneity [31] and that the analysis of multiple cores per tumor specimen might enhance the representativity of TMA studies [32]. These authors suggest that there might exist a better concordance of large section findings with tissue microarrays data, if 3–4 cores were analysed per cancer on comparison to the use of only one core. However, these suggestions were based on the assumption that significant heterogeneity may exist within the tissue represented by a standard 3 × 4 cm paraffin block, and that large section analysis is the method of choice to estimate tumor heterogenity. In our opinion, this assumption is disputable, since previously, data have shown that the TMA format is generally superior over large section studies to analyse potential correlations between molecular markers and the clinical outcome [33] and that there were found high significances between biomarkers and clinical outcome irrespectively if the three tissue cores were analyzed separately, or if a combined result was generated from the three cores [34]. These data demonstrate that the use of multiple cores in a TMA does not necessarily enhance the ability to identify significant relationships between biomarkers and tumor phenotype and/or prognosis. Moreover, these data underline the robustness of IHC microtissue array studies for analysis of correlations of molecular markers with clinico-pathological features of cancer specimens.

In summary, our data show that decreased RBM3 expression is associated with unfavourable esophageal cancer phenotype but is unrelated to prognosis of patients. Therefore, RBM3 cannot be considered as a clinically relevant prognostic biomarker in esophageal cancers.

## Conclusions

In summary, the present study shows that decreased RBM3 expression is associated with unfavourable oesophageal cancer phenotype, but does not predict patients’ prognosis.

## Abbreviations

EAC: Esophageal adenocarcinoma
ESCC: Esophageal squamous cell cancer
IHC: Immunohistochemistry
RBM3: RNA binding-protein 3
TMA: Tissue microarray
